# Ion Channels in Epithelial Dynamics and Morphogenesis

**DOI:** 10.3390/cells10092280

**Published:** 2021-09-01

**Authors:** Ankit Roy Choudhury, Jörg Großhans, Deqing Kong

**Affiliations:** Department of Biology, Philipps University, 35043 Marburg, Germany; ankit.roychoudhury@biologie.uni-marburg.de (A.R.C.); grosshan@uni-marburg.de (J.G.)

**Keywords:** *Drosophila*, epithelial cells, morphogenesis, mechano-gated ion channels, calcium ion

## Abstract

Mechanosensitive ion channels mediate the neuronal sensation of mechanical signals such as sound, touch, and pain. Recent studies point to a function of these channel proteins in cell types and tissues in addition to the nervous system, such as epithelia, where they have been little studied, and their role has remained elusive. Dynamic epithelia are intrinsically exposed to mechanical forces. A response to pull and push is assumed to constitute an essential part of morphogenetic movements of epithelial tissues, for example. Mechano-gated channels may participate in sensing and responding to such forces. In this review, focusing on *Drosophila*, we highlight recent results that will guide further investigations concerned with the mechanistic role of these ion channels in epithelial cells.

## 1. Introduction

Epithelial cells constitute one of the four general tissue types. Epithelial tissue does not only cover the whole organism as such but also wraps all the visceral organs. Epithelial cells are polarised, i.e., the cortical proteins and organelles are differentially distributed. Tight and septate junctions segregate the apical side from the basal within a typical epithelial sheet, forming a diffusion barrier. In contrast, epithelial cells differ internally within the plane of a tissue sheet, establishing planar cell polarity [1]. Epithelial tissues are meant to undergo a series of morphodynamic and functional changes in the course of development.

**Figure 1:**
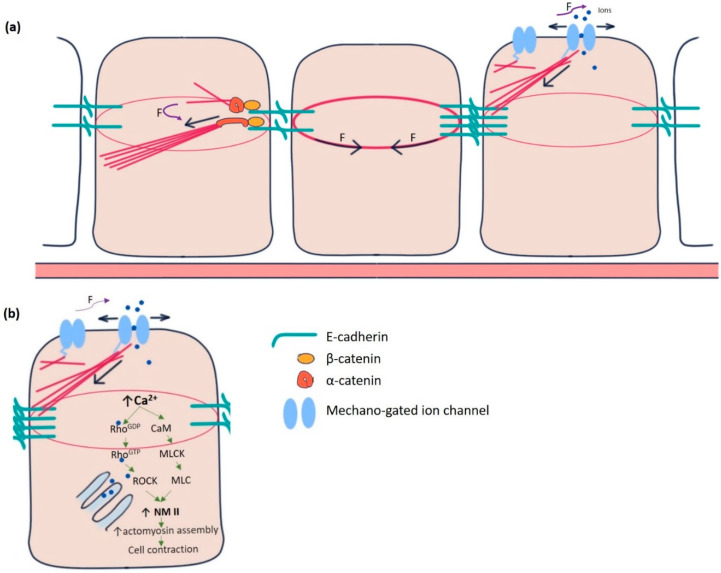
Mechanotransduction machinery in epithelial cells. (**a**) The E-cadherin-catenin complex is the key to adherens junction-mediated mechanotransduction. Pulling force (F) due to the contractile actin ring of the cell at the centre promotes a force-induced conformational change in α-catenin from closed to open conformation in the cell at the left. α-catenin at its open conformation binds with actin-binding protein vinculin and acts as an actin nucleator. Mechano-gated channels (in the cell at right) can sense forces directly from the membrane or the cytoskeleton. In response, they change conformation from closed to open state and allow the ions to flow in and out of the cell. (**b**) Increased intracellular Ca^2+^ concentration in epithelial cells modulates the actomyosin assembly by activating non-muscle myosin II (NMII), the principal effector molecule. CaM, Calmodulin, MLCK, myosin light chain kinase, MLC, Myosin light chains, ROCK, Rho-associated kinase.

Individual epithelial cells communicate chemically or mechanically to promote tissue morphogenesis, remodelling, and pattern formation [2]. Chemical coordination is widespread and long-lived but time-consuming. On the contrary, mechanical communication is almost instantaneous, although spatially limited, between neighbouring cells [3]. It is remarkable how thousands of epithelial cells work in unison to polarise their force-generating types of machinery and remodel their contacts during such tissue-scale changes. The adherens junctions and cytoskeleton mediate mechanical communication. At the centre of adherens junctions, E-cadherin-catenin complexes constitute the mechanical link between neighbours by the Ca^2+^-dependent homotypic trans-binding of two extracellular domains. α-catenin links adherens junctions to the cytoskeleton [4]. Mechanical forces from the actin cytoskeleton prompt the conformational change in α-catenin from its closed to open state, facilitating actin-binding protein vinculin to interact with α-catenin. The link between E-cadherin clusters and the cytoskeleton is reinforced and strengthened in this manner (Figure 1a) [5,6]. Besides adherence junctions, integrin-rich focal adhesion sites at the basal domain of epithelial cells establish mechanical reciprocity between the viscoelasticity of the ECM and the traction force exerted by the cell [7]. Integrins are essential for epithelial polarisation around epidermal wounds in Drosophila, eventually leading to the closure [8].

Yet another category of mechanosensitive proteins, called mechano-gated ion channels (MGCs), had long been elusive for functioning in epithelial cell communication (Figure 1a). During the last two decades, several researchers gradually pointed out the existence and importance of these channels in the spectrum of model organisms, including *Drosophila* [9,10,11]. In *Drosophila,* such channels were initially identified in the sensory neurons, primarily involved in proprioception, nociception, hearing, locomotion, etc. [12,13]. These channels remain in “open” or “closed” conformational states. The switching between these states is regulated by mechanical force exerted by the plasma membrane or the cytoskeletal proteins [14]. Being in the open state, MGCs exhibit permeability to the ions, such as Ca^2+^, K^+^, Na^+^, and Cl^−^, which act as effector molecules to induce various signalling pathways. For example, Ca^2+^ has been shown to promote epithelial tight junction remodelling by activating RhoA in *Xenopus* embryonic epithelium (Figure 1b) [15].

This review will focus on *Drosophila* and highlight some of the promising new developments that have paved the way to investigate further the mechanistic role of these ion channels in epithelial cells with greater detail.

## 2. Epithelial Cells in *Drosophila*

**Figure 2:**
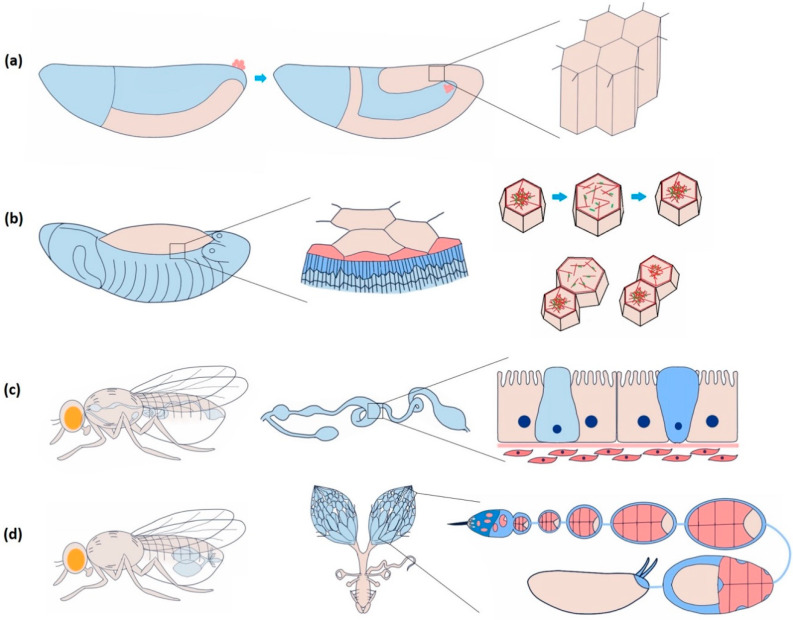
Dynamic epithelial tissues during morphogenesis and homeostasis in *Drosophila*. (**a**) Highly dynamic columnar epithelial cells of the germband during germband extension. The frequency of Ca^2+^ spikes increases during the first fast phase of germband extension, though the correlation with cell and junction dynamics are still unclear. (**b**) Periodically oscillating squamous epithelial cells of amnioserosa before dorsal closure. Neighbouring cells are either positively (contract and relax together) or negatively (when one contracts, the other relaxes and vice versa) coupled. Negative coupling maintains the tissue area and integrity, whereas positive coupling induces closure. Ca^2+^ influx is sufficient to induce cell contraction guided by actomyosin assembly. (**c**) *Drosophila* midgut comprises a diverse group of cuboidal epithelial cells: enterocyte with microvilli for absorption, intestinal stem cell (light blue) for regeneration and proliferation, and enteroendocrine cell (dark blue) for secretion. Ca^2+^ induces proliferation of the intestinal stem cells. Piezo-mediated Ca^2+^ is attributed to initiating the differentiation of the enteroendocrine cells. (**d**) The *Drosophila* ovary consists of a series of developing egg chambers. A dense monolayer of somatic epithelial cells called follicle cells surrounds a single oocyte and 15 supporting nurse cells in each egg chamber. Egg chamber elongation largely depends on the asynchronous oscillation of these cells, which Ca^2+^ regulates.

In *Drosophila*, the epithelial epidermis undergoes a series of spatially defined morphogenetic movements from gastrulation onwards, including tissue invagination, collective cell migration, convergent extension, dorsal closure, tube formation, head involution, etc. [16]. Coordination among the epithelial cells is necessary to ensure tissue integrity for the morphogenetic events to occur seamlessly [17]. Endodermal cell masses from both the ends of the *Drosophila* embryo collectively migrate along the visceral mesoderm and merge to form the continuous gut epithelium [18]. Yeast ingestion-induced stretching of mature gut epithelium causes *yki* (yorkie)-mediated proliferation, lacking which the tissue may undergo atrophy [19]. Coordinated asynchronous oscillations of the follicle cells in the *Drosophila* ovary are essential for egg-chamber elongation [20]. The E-cadherin-based mechanical connection between border and nurse cells is necessary for border cell migration in the egg chamber [21]. In Figure 2, we exemplify a few of the dynamic epithelial tissue types and morphological characteristics of the cells involved.

## 3. Calcium Ion in the Epithelium

The importance of calcium signalling in epithelial morphogenesis has been found to be crucial in various model organisms. The convergent extension is defective under the inhibition of calcium signalling in Zebrafish and *Xenopus*. In contrast, the experimentally induced increase in calcium ion (Ca^2+^) concentration triggers gastrulation in *Echinoidea*, neural fold formation in *Ambystoma,* and egg chamber elongation in *Drosophila* [22,23,24,25,26]. Two patterns of Ca^2+^ activity have been reported in *Drosophila* early embryos: (a) Ca^2+^ waves that are spontaneous, repetitive, and are often followed by a wave of tissue contraction, and (b) Ca^2+^ spikes that arise stochastically in a single cell or a group of few cells and are transient [27]. In the *Drosophila* wing disc, intracellular Ca^2+^ transients act as a signal integrator and decrease over time as the wing disc matures [28,29]. Increased intracellular Ca^2+^ corresponds to intestinal stem cell proliferation in *Drosophila* by regulating calcineurin and CRTC (CREB-regulated transcriptional co-activator) [30]. In processes like wound healing, Ca^2+^ waves help build rapid communication across many cells [31,32]. Ca^2+^ spikes are proven to have a close connection with Wnt signalling, the inhibition of which leads to decreased spike activity and eventually morphogenetic impairment in the developing embryo [33,34,35]. Such waves and spikes temporally coincide and thus hold the potential to regulate various morphogenetic events like dorsal closure, cuticle formation, and head involution [27,35].

The reciprocity between cell contraction and adherens junction-mediated force transduction to the neighbouring cells contributes to emergent tissue behaviours like folds and furrows formation [36]. Intracellular Ca^2+^ has long been a principal regulator of contraction in many cell types, including muscle cells, stromal fibroblasts, and epithelial cells in culture [37,38,39,40]. Experiments in *Drosophila* embryos pointed out the importance of intracellular Ca^2+^ to induce contractility in amnioserosa cells during dorsal closure, neural tube closure, and neural plate folding [41,42,43,44]. Ventral furrow formation during gastrulation and contraction of amnioserosa cells during dorsal closure revealed the “ratchet” mechanism caused by a pulsatile cortical network of medioapical actomyosin [45]. The contractility in such non-muscle cells is driven by non-muscle myosin II (NM II), primarily regulated by Rho-ROCK signalling [46,47,48]. In the follicle cells of the *Drosophila* blade, intracellular Ca^2+^ seems to control the basal concentration of NM II. Chelating cytosolic Ca^2+^ by BAPTA reduces basal NMII. The effect can be reversed by adding ionomycin, driving Ca^2+^ influx [26]. Intracellular Ca^2+^ can also directly form a complex with tetravalent calmodulin protein, activating myosin light chain kinase (MLCK) that activates the regulatory light chain of NM II (Figure 1b) [49,50].

The amnioserosa is a monolayer of 150–200 autonomously oscillating squamous epithelial cells covering the dorsal opening of developing *Drosophila* embryos at stages 13–15 (Figure 2b) [51,52]. Inducing rapid Ca^2+^ bursts by uncaging intracellular Ca^2+^ can trigger amnioserosa cell contraction in single-cell resolution by activating NM II, wherein Ca^2+^ is reported to be linked at the position of ROCK (Rho-associated kinase) in the Rho-ROCK pathway [53]. Besides directly phosphorylating the myosin II regulatory light chain (RLC), ROCK also prevents the dephosphorylation of NM II by inhibiting protein phosphatase I (PP I), stabilising the activated NM II [54]. Constitutive activation of MLCK in the entire amnioserosa results in the overall rounding of the cells. Expression in individual cells triggers premature apical constriction [55]. In 3T3 fibroblast cells, ROCK is more centrally localised, whereas MLCK is localised more towards the periphery [50]. This localisation bias has to do with the spatially differential stability of the actomyosin structure within a cell. It could be investigated further to confirm such an argument in epithelial cells.

## 4. Mechanosensitive Ion Channels in the Epithelium

**Figure 3:**
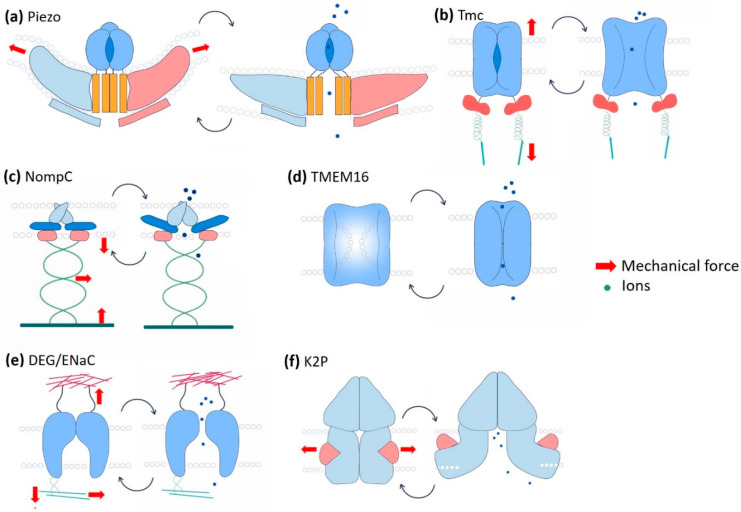
Schematic representation of mechano-gated channels and their gating mechanisms. (**a**) The Piezo channel is supposed to form a caveolae-like depression in the membrane. It is directly gated by membrane tension. Directional membrane tension flattens the channel to allow Ca^2+^ influx. (**b**) The transmembrane Channel-like Protein (Tmc channel) forms complexes with a group of other proteins that bring extracellular mechanical inputs. Intracellular Tmc is connected to the cytoskeleton by ankyrin and calcium-binding proteins. The channel is gated via the tension mediated by the tethering proteins from both extra and intracellular interfaces. (**c**) No Mechanoreceptor Potential C (NompC) has a substantially long (29 ankyrin repeats) cytoplasmic domain that interacts with the microtubules. The gating of NompC depends on the deflections of the ankyrin repeat helices mediated by the cytoskeleton components. (**d**) Transmembrane protein 16 (TMEM16) is a calcium-dependent chloride channel (CaCC). The dual function of TMEM16 as a lipid scramblase and as an ion channel makes it unique. These two functions are mutually exclusive and depend on the membrane tension-mediated conformational change of the protein. (**e**) The epithelial sodium channel (ENaC) interacts with both extracellular and intracellular proteins. Hydrostatic pressure, membrane stretch, and shear forces are the key mechanical cues that determine the gating of the channel pore. (**f**) The two pore domain K^+^ channel (K2P), in its closed state, is blocked by the lipid. Membrane tension releases the lipid blockade and allows the hinge-like bending of the transmembrane domain and eventually ion flux.

Not only are highly specialised sensory cells involved in hearing and proprioception, but potentially almost every eukaryotic cell can sense the force from its milieu via the conformational changes of membrane-bound proteins or protein complexes, so-called mechanosensors. These mechanosensors can detect and transduce the external mechanical signal into a cell. Junctional molecules, cytoskeletal proteins, G-protein coupled receptors, and mechanosensitive ion channels (MSCs) constitute a wide range of mechanosensors [56,57]. MSCs are evolutionarily ancient, pore-forming integral membrane proteins present in literally every living organism from archaea to bacteria to eukaryotes [58]. While in an open state, they allow ions such as Ca^2+^, Na^+^, K^+^, and Cl^−^ to flow into and out of cells. The gating behaviour, i.e., the transition from closed to open conformation of MSCs, is regulated either by forces parallel to the plasma membrane (membrane tension model) or by forces applied by the associated cytoskeletal or extracellular matrix proteins (tether model) [14,59]. Channels are considered mechanically gated if a specific stimulus is immediately followed by the ion flux, at least faster than any other known second messenger and if knockdown of their expression leads to a loss of mechanosensory response [60]. In the following, we will discuss several mechano-gated ion channels, namely, Piezo, transmembrane Channel-like Protein (Tmc), No Mechanoreceptor Potential C (NompC), Transmembrane protein 16 (TMEM16), epithelial sodium channel (DEG/ENaC), and two pore domain K^+^ channel (K2P), in *Drosophila* epithelial morphogenesis (Figure 3).

### 4.1. Piezo Channel

Since Piezo1/2 was discovered in 2010, many investigations have been conducted to reveal the structure and function of these novel groups of cation channels in eukaryotes [61]. Piezo proteins are approximately 2500 amino acids long and possess numerous transmembrane domains [62]. A cryo-EM study has revealed that Piezo consists of a central cap, three peripheral extracellular blade-like domains, and three long beams on the intracellular side (Figure 3a) [63]. Purified and membrane-incorporated Piezo1 allows cation influx across the membrane while subject to mechanical stress [64]. Piezo1 preferentially allows calcium influx in response to stimulation in whole-cell or outside-out patch-clamp recording [65]. GsMTx4, a peptide isolated from the tarantula spider and a known modifier of MSC gating, blocks Piezo1 activity [66]. A small molecule called Yoda1, on the contrary, can trigger Piezo1 and Piezo1-mediated calcium influx even in the absence of a mechanical stimulus [67]. Similar to Piezo1, Piezo2 also acts as a Ca^2+^-sensitive mechano-gated channel. Piezo2-mediated Ca^2+^-influx is reported to activate RhoA, which controls the assembly and orientation of stress fibres and focal adhesions [68].

Recent studies revealed the involvement of the Piezo channel in epithelial cell homeostasis using MDCK cells in culture. Piezo1 detects and transduces epithelial cell stretch at low-cell-density areas, resulting in cell division [69]. Genetic knockdown of Piezo1 hinders homeostatic cell extrusion in developing zebrafish epidermis, leading to the formation of epithelial cell clusters [70]. Piezo channels are expressed across the body in many different types of epithelial cells, subject to compressive and shear stresses, for example, vascular endothelial cells, mammary epithelial cells, urinary bladder cells, pancreatic acinar cells, and so on [71]. Piezo1 knockout causes embryonic lethality of E14.5 mice due to impaired vasculogenesis [72]. Piezo2 has recently been discovered in the human enteroendocrine cell (EEC) population [73].

The *Drosophila* genome contains two Piezo genes: *Piezo*, an ortholog, and *piezo-like (Pzl)*, a homolog of *Piezo* gene families. *Piezo* knockout flies are viable and fertile, and it seems not to induce major developmental defects. *Piezo* knockout larvae show severely reduced behavioural responses to noxious mechanical stimuli [12]. *Pzl* is functional in proprioceptive chordotonal neurons of *Drosophila* larvae. Loss of *Pzl* severely affects the locomotion and body gesture control in the larvae and can be rescued by the expression of human or mouse Piezo1 [74]. *Drosophila* midgut, analogous to the stomach and small intestine in vertebrates, has a distinct population of intestinal stem cells. These cells commit to becoming secretory EECs under the influence of low Notch signalling, constituting 1% gut epithelial cells. Distension of the gut by mechanical forces triggers the differentiation of Piezo^+^ EEC precursors into Piezo^+^ EEC. He et al. [75] proposed that Piezo-mediated Ca^2+^ influx and Notch inhibition were sufficient to drive the EEC differentiation through a series of experiments. On the other hand, ERK signalling drives EEC cell proliferation but not differentiation [75,76]. These findings raise open questions like whether these pathways are mechanically interlinked and Piezo’s possible implication in human gastrointestinal pathologies.

### 4.2. Transmembrane Channel-like Protein (Tmc)

The TMC family comprises integral membrane proteins of a ~115 amino acid-long TMC domain that starts with a highly conserved “CWET” signature sequence (Figure 3b) [77]. Vertebrates have eight TMC proteins encoded by *TMC1-8* genes [78,79]. TMC4 is expressed in the kidney, small intestine, and colon epithelia. It is proposed to form a Ca^2+^-dependent Cl^-^ channel [80]. TMC6 and TMC8 are associated with zinc transporters in keratinocytes [81]. Mechanosensory functions of TMC proteins are not conclusive except for TMC1 and TMC2, which are found to form the core of a multimeric mechanosensitive complex in auditory hair cells and lateral line organs of fish [82,83]. A sound wave creates deflections of the “hair bundle,” a cluster of actin-rich stereocilia located at the apical surface of the hair cells. Extracellular protein filaments called “tip links” transmit mechanical distortions to the MSCs at the stereocilia tips [84]. Tip links interact with protocadherin-15 (PCDH15) homodimers, transmembrane, and cytoplasmic domains, further interacting with the TMC proteins [85,86]. In *C*. *elegans*, ankyrin is found to tether TMC channels intracellularly like a spring via Calcium and Integrin Binding protein, CALM-1 [87].

Both TMC1 and TMC2 are highly selective to Ca^2+^; however, TMC2 has ~3-fold higher selectivity than TMC1 [88]. In mice, TMC2 is expressed from birth to postnatal day 10 and then declines to zero, followed by expression of TMC1 that continues throughout life [89]. The upsurge of TMC1 localisation at the tips of hair cell stereocilia parallels with the onset of auditory function [88,90]. TMC1 and TMC2 seem to have mutually distinct physiological functions, as the expression of TMC2 cannot compensate for the loss of expression of TMC1 in auditory hair cells. Zebrafish neuromast cells along the lateral line organ can be classified into three groups based on their differential reliance on the TMC2b channel for mechanotransduction [83].

In *Drosophila*, the only *Tmc* gene translates to the protein. The sequence is highly conserved with *Tmc* family members across different organisms. *Tmc* is expressed in Class I da, Class II da, and bd sensory neurons. *Tmc* mutant larvae have defects in locomotion, and adults show difficulty in food texture sensation [91]. The roles of Tmc in epithelial morphogenesis have not been studied thoroughly yet. Our work indicates an essential function of *Tmc* in homogenous tension distribution across the oscillating amnioserosa cells and maintaining the synchronisation of neighbouring cells [92]. Moreover, *Tmc* null embryos exhibit a significant reduction in Ca^2+^ influx in response to wounding or tissue damage. It is worth investigating the molecular mechanism and the direct interacting partners of Tmc in the future.

### 4.3. No Mechanoreceptor Potential C (nompC)

*nompC* is the only member of the TRPN family that comes under a large superfamily of characterised or putative MSC proteins, known as transient receptor potential (TRP) channels. The TRP superfamily consists of more than 30 cation channels, most of which are permeable to either Ca^2+^ or Mg^2+^ [93]. The gene *nompC* was first identified in *Drosophila* in a screen for mechanoreceptive mutants with defects in mechanosensory physiology. Loss-of-function point mutations of *nompC* abolish the mechanosensory transduction current. Various nonsense and missense point mutations of *nompC* cause a series of defects in *Drosophila*, such as hearing impairment, locomotion, gentle touch sensation, adaptive response to mechanical stimuli, and food texture sensation [13]. The structure of NompC was described in 2017 by single-particle cryo-electron microscopy. NompC protein contains a short transmembrane neck domain and a distinctive 29 ankyrin repeats-long helical cytoplasmic domain (Figure 3c). NompC ankyrin repeats interact with microtubules and is implicated as a rope to convey force from the cytoskeleton to the channel, thus controlling the gating [94,95]. Most TRP channels are reported to be permeable for monovalent cations like Na^+^ and K^+^ and divalent cations like Ca^2+^ [96,97,98]. Ca^2+^ influx is proposed to change the state of motor proteins that, in turn, adjust cellular tension [97].

A recent finding demonstrates that *nompC* point mutations are attributed to defective dorsal closure phenotypes in the *Drosophila*, such as irregular purse string and increased embryonic lethality [99]. Furthermore, induced expression of NompC constructs with truncated ankyrin repeats in amnioserosa leads to defects in dorsal closure, like failure to organise an actomyosin purse string, loss of leading-edge cell elongation, and so on [99]. Taking these results into account, it is worth investigating the physiological relevance of *nompC* in amnioserosa and the underlying mechanism of NompC mechanotransduction that leads to the rapid tissue-scale transmission of multidirectional forces.

### 4.4. Transmembrane Protein 16 (TMEM16)

Though in the early 1980s, Ca^2+^-activated Cl^−^ channel (CaCC) activity was observed in frog oocytes [100,101], three groups independently developed their molecular identity in 2008. The gene encoding TMEM16A is responsible for the Cl^−^ currents in response to increased intracellular Ca^2+^ concentration [102,103,104]. The TMEM family includes CaCCs like TMEM16A and TMEM16B. Most family members are Ca^2+^-activated scramblases, facilitating bidirectional diffusion of lipids between membrane leaflets [105]. Cryo-EM revealed that TMEM16A is a dimeric channel. Each subunit includes 10 transmembrane segments (Figure 3d) [106]. In silico analysis demonstrates an evolutionary connection of TMEM with a Ca^2+^-permeable stress gated cation channel (CSC), Tmc, etc. [107]. In human and mouse bile duct epithelial cells or cholangiocytes, shear stress is shown to activate TMEM16A-mediated Cl^−^ transport, a process dependent on extracellular ATP and intracellular Ca^2+^. The mechanical stimuli do not directly regulate the channel’s gating; rather, it depends on the rate-driven delivery of ATP to the membrane (shear rate) [108].

In *Drosophila*, TMEM16A or *subdued* is found in non-excitable epithelial cells and implicated in host defence [109,110,111]. An absence of TMEM16 is marked by dysmorphic epithelial organisation in the trachea, oesophagus, and kidney of mouse embryos [112]. TMEM16A is essential to maintain a critical level of cytoplasmic Cl^−^. Cytoplasmic Cl^−^ is necessary to regulate microdomain partitioning of PI(4,5)P_2_, endocytic trafficking, and recycling endosome, ensuring membrane supply during ciliogenesis and junctional remodelling [112]. TMEM16A seems to have a bunch of activating agents working in parallel. It also appears to induce various signalling pathways upon activation. Hence, it is worth investigating the molecular association and the procedure of mechanical induction of TMEM16A more thoroughly.

### 4.5. DEG/ENaC Channels

Monovalent cations like sodium ions (Na^+^) are implicated in epithelial homeostasis and morphogenesis [113,114]. Recent studies have found multiple known sodium channels to be mechanically gated. Na_v_1.5, a voltage-sensitive sodium channel in the human heart and gut, is activated by membrane stretching. Mutations of this channel disrupt the mechanical sensitivity of gut epithelial cells, resulting in abdominal pain syndrome and irritable bowel syndrome [115]. Epithelial sodium channel (ENaC) mediates passive sodium transport at the apical domain of many different epithelial cell types: kidney, lungs, skin, colon, and reproductive tract [116,117,118,119,120]. ENaC can be activated by shear force across organisms, especially in vascular endothelium, to maintain its tonicity [121]. Shear forces can be transduced by the N-glycosylated extracellular domain of ENaC tethering with extracellular matrix (ECM) [122]. ENaC directly interacts with spectrin, ankyrin, actin cytoskeleton, and actin-associated proteins [123,124,125]. Cellular responses to hydrostatic pressure differences and membrane stretch depend on such interactions [126]. Degenerin (Deg), *C. elegans*-specific ENaC, gained function mutations resulting in degenerations like swelling, vacuolation, and apoptosis [127]. Studying mechanoreceptor currents reveals activation of DEG channels in response to gentle and nociceptive mechanical stimuli [128].

Ripped pocket (Rpk) and Pickpocket (Ppk) were identified as two novel ENaC proteins in 1998 in *Drosophila*. *rpk* transcripts are abundant in early-stage embryos and adult ovaries, whereas *ppk* is only expressed in sensory neurons in late-stage embryos [129]. It points to the potential functions of *rpk* during early embryonic development. Rpk localises in patches at the apical surface but not at the junctions of amnioserosa cells. Knockdown of *rpk* in amnioserosa causes elongation failure of the lateral epidermis. *rpk* mutant embryos show impaired epitheliogenesis, including defective germband extension, dorsal closure, head involution, and consequent lethality [41]. *rpk* has recently been implicated in depolarising the membrane potential of anterior epithelial cells during imaginal disc development. Rpk expression in these cells depends on Hedgehog (Hh) signalling. Suppression of *rpk* leads to a reduction in depolarisation of anterior cells and a disruption in compartmentalisation between anterior and posterior cell populations [130].

### 4.6. K2P Channels

The K2P (two pore domain K^+^ channel) family of channels are potassium (K^+^) channels made of a dimeric assembly of two subunits, each containing four transmembrane domains [131]. The channel was first identified in yeast [132]. Later on, 15 channels of the K2P family were discovered in mammals by sequence homology screening of the pore domain region [133]. Out of these 15 channels, three channels appear to be mechanically gated, namely, TREK-1 (TWIK-related K^+^ channel 1), TREK-2, and TRAAK (TWIK-related arachidonic acid-stimulated K^+^ channel). K^+^ currents through these channels can be mechanically induced by membrane stretch in both in vitro and in vivo systems [134,135,136]. The *Xenopus* oocyte expressing TREK-1 channel shows induced channel activity upon mechanical stress like membrane stretch, osmotic swelling, and shear stress [137]. TREK-1 also induces actin cytoskeleton remodelling and colocalises with ezrin in filopodia-like structures [138]. Studies in the human alveolar epithelial cell line reveal that TREK-1 regulates cell deformability by cytoskeletal remodelling and cell detachment following mechanical stretch [139]. However, whether the cytoskeletal interaction is responsible for channel gating remains unclear.

TREK-1 is essential for the proliferation of the human endometrial epithelial cell line [140]. Deficiency of TREK-1 ortholog *sandman* in *Drosophila* causes cardiac fibrosis and diastolic dysfunction [141]. Decreased expression of *Drosophila* TREK-1 homolog *Ork-1* (Open rectifier potassium channel 1) has been found to modulate learning and sleep behaviour [142]. Cardiac-specific inactivation of *Ork-1* causes increased heart rhythm [143]. The gating of K2P channels is regulated by the transmembrane domain (TM2.6), more specifically by a single amino acid (aspartate) residue in this domain both in the vertebrates and invertebrates like *Drosophila* [144,145]. The role of K2P channels is still not clear in epithelial cells in *Drosophila*. Promising studies from other model organisms and direct membrane-stress-induced mechano-gating like the Piezo channel held K2P channels worth investigating in *Drosophila*, especially in the context of epithelial morphogenesis where mechanical forces are very obvious and dynamic.

## 5. Concluding Remarks

Genome-wide studies have revealed the expression of mechano-gated and mechano-sensitive ion channels outside of their typical cell types within the nervous system and neurosensory organs. Despite their presence, a physiological function of these channels has remained elusive in epithelia. The new-generation microscopy techniques and high-throughput quantitative image analysis have improved the tractability of tissue-wise cellular motions [146,147]. Hence, the changes in epithelial morphodynamics and the underlying biomechanical perturbations inflicted by the mutants of the mechano-gated channels can be studied in depth.

TRP channels like *Trp1* are reported to severely affect the dorsal closure in *Drosophila* [41]. Although Trp1 is not yet shown to be mechanically gated, it can disrupt the force orchestration in and around amnioserosa tissue. Both mouse TRPM7 and its ortholog *Trpm* in *Drosophila* are shown to be responsible for Ca^2+^ influx during egg activation [148]. Knockdown of *Trpm* results in decreased intracellular calcium and impaired actomyosin cable formation in post-wounding *Drosophila* pupal notum epithelium [149]. TRPM7 is activated by mechanical triggers, but whether TrpM in *Drosophila* responds the same way in response to mechanical cues during ovulation remains unclear [148]. Two more *Drosophila* proteins, Brivido-1 (Brv1) and Tmem63, reportedly form mechano-gated ion channels. However, their roles in mechanotransduction are not clear. Brv1 coexpresses NompC in larval class III da neurons, where it seems to facilitate mechanoactive NompC currents in gentle touch sensation [150]. Tmem63 needs a relatively strong mechanical stimulus to get activated [151]. The expression and mechanosensory activities of these ion channels in the context of epithelial homeostasis and morphogenesis need to be addressed.

By combining the ions, cytoskeleton, and junctional proteins, mechanosensitive ion channels became a fascinating group of ion channels in the epithelium. For example, NompC was revealed to link to microtubules for channel opening, Piezo1 shows an essential function for Rho signalling, and the adherence junction is a defect in the TMEM16 mutant. These phenomena are awe-inspiring. It is worth investigating in the future whether (1) mechanosensitive ion channels interact with junctional proteins physically, (2) mechanosensitive ion channels coordinate with junctional proteins during mechanotransduction, and (3) mechanosensitive ion channels open via the mechanical forces from junction proteins.
